# Low Use of Standard-of-Care Antiparasitic Drugs and Increased Estimated Outpatient Payments for Treating Schistosomiasis in the United States, 2013–19

**DOI:** 10.4269/ajtmh.22-0254

**Published:** 2022-08-22

**Authors:** Heesoo Joo, Brian A. Maskery, Jonathan D. Alpern, Rebecca J. Chancey, Michelle Weinberg, William M. Stauffer

**Affiliations:** ^1^ Division of Global Migration and Quarantine, Centers for Disease Control and Prevention, Atlanta, Georgia;; ^2^ HealthPartners Institute, Bloomington, Minnesota;; ^3^ Department of Medicine, University of Minnesota, Minneapolis, Minnesota;; ^4^ Division of Parasitic Diseases and Malaria, Centers for Disease Control and Prevention, Atlanta, Georgia;; ^5^ Center for Global Health and Social Responsibility, University of Minnesota, Minneapolis, Minnesota

## Abstract

Drug utilization and payment estimates for standard-of-care treatment of schistosomiasis have not been reported previously in the United States. This study estimates the utilization of praziquantel (standard-of-care drug) among patients with schistosomiasis and outpatient payments among those who were treated with praziquantel, and investigates the factors associated with praziquantel use from 2013–19 using IBM’s MarketScan^®^ Commercial Claims and Encounters database. Claims data showed that only 21% of patients with schistosomiasis diagnoses were treated with praziquantel. The mean total drug payments per patient treated with praziquantel increased from $110 in 2013–14 to $612 in 2015–18 (*P* < 0.01), and use decreased. These factors, including residing in a rural area, having a documented *Schistosoma haematobium* infection, or having a first schistosomiasis diagnosis in 2015–16, were associated with a decreased likelihood of patients receiving standard-of-care treatment. Policy solutions to exorbitant drug pricing, and better awareness and education among healthcare providers about schistosomiasis—especially those practicing in rural areas with high immigrant populations—are needed.

Human schistosome infections in the United States are rare and affect immigrants and refugees disproportionately. Untreated intestinal schistosomiasis can lead to chronic hepatic disease, including fibrosis and portal hypertension, and urogenital schistosomiasis has been associated with bladder cancer.^1^ Both forms can cause ectopic granulomatous lesions and have symptoms that can lead to invasive medical examinations.^2^^,^^3^ Praziquantel is the drug of choice for the treatment of schistosomiasis (40 mg/kg/day in two divided doses for 1 day for *Schistosoma mansoni*,* Schistosoma haematobium*, or *Schistosoma intercalatum* in the United States).^4^ The nominal average wholesale price (AWP) per unit package (3,600 mg) of Biltricide^®^ (Bayer HealthCare Pharmaceuticals Inc., Montville, NJ), the brand name of praziquantel, was $86 in January 2009 and increased gradually to $131 in 2014.^5^ The AWP then increased dramatically by more than 350% in February 2015 to around $600 per unit package. The AWP of Biltricide^®^ did not change between February 2015 and May 2021.

The utilization of praziquantel and treatment costs associated with schistosomiasis in the United States have not been reported previously. This study estimated the praziquantel use among privately insured patients with schistosomiasis diagnoses, and examined factors associated with untreated schistosomiasis by comparing characteristics of patients with schistosomiasis diagnoses who were treated with praziquantel and characteristics of untreated patients, by using a commercial claims database in the United States. The impact of the increased price of praziquantel on outpatient payments for patients with a schistosomiasis diagnosis in the United States was measured, and trends in non-drug and drug payments for outpatient visits over time were reported.

Patients with a first diagnosis of schistosomiasis in the IBM^®^ MarketScan^®^ Commercial Claims and Encounters database (IBM Watson Health, Ann Arbor, MI) from January 1, 2013 to December 31, 2019 were selected using an online tool (Truven Health MarketScan Treatment Pathways).^6^ Patients with schistosomiasis diagnoses were identified using *International Classification of Diseases, Ninth Revision, Clinical Modification* (ICD-9-CM) codes 120.0, 120.1, 120.8, and 120.9; and ICD-10-CM codes B65.0, B65.1, B65.8, and B65.9.

Patients with a first diagnosis of schistosomiasis during 2013–19 were included if they met the following criteria: were continuously enrolled in private insurance between 30 days before and 90 days after the first diagnosis, had a single diagnosis code for a parasitic disease (i.e., patients with co-infections, including ascariasis, hookworm, trichuriasis, and strongyloidiasis, were excluded), and made only outpatient visits associated with schistosomiasis.

Among these selected patients, we compared the sample characteristics of those who were treated with praziquantel to those who were not treated with praziquantel between 30 days before and 90 days after the first diagnosis. This time frame was chosen because praziquantel may be prescribed before the infection is confirmed when clinical suspicion is high. Logistic regression analysis was used to assess the association between sample characteristics and the use of praziquantel. Regression analyses included gender (male versus female [reference]), age (> 40 years versus ≤ 40 years old [reference]), geographic region (rural versus urban [reference]), number of outpatient-visit days associated with schistosomiasis (1 day [reference] versus ≥ 2 days), insurance type (capitated versus non-capitated [reference]), species of schistosome (*S. haematobium*,* S. mansoni*, or a combined category including “other specified or unspecified schistosomiasis”; i.e., ICD-9-CM codes 120.8, and 120.9; ICD-10-CM codes B65.8 and B65.9 [reference]), and period (2013–14 [reference], 2015–16, and 2017–19). The number of outpatient-visit days was the total number of days with outpatient visit claims with a diagnosis code for schistosomiasis between 30 days before and 90 days after the first diagnosis. For instance, if a patient had two outpatient visits in 1 day, it was reported as a single outpatient-visit day. The reference period, 2013–14, represented the period before the AWP increased.

A subset of patients who were treated with praziquantel and for whom payment data were available was created by excluding patients who 1) had capitated insurance plans, 2) were not treated with praziquantel, 3) were first diagnosed with schistosomiasis in 2019, or 4) had reported total payments, including both outpatient visits and drug payments, below the 1st or above the 99th percentile to eliminate outliers. Those who were first diagnosed with schistosomiasis in 2019 were excluded because payment data for the MarketScan^®^ Commercial Claims and Encounters database were not available for that year. Mean and 95% confidence intervals (95% CIs) of drug and non-drug outpatient payments (e.g., physician fees, laboratory test fees etc.) between 30 days before and 90 days after the first diagnosis of schistosomiasis by period were reported. Total payments were subdivided into insurance and patient out-of-pocket payments. The changes in payments over time were tested for statistical significance using Welch’s *t*-test with unequal variances. The payments in 2013–14, the period before the substantial AWP change, were treated as the reference for *t*-tests. *P* values less than 0.05 were considered statistically significant. All analyses were performed using Stata/SE (StataCorp LLC, College Station, TX, version16.1).

Of the 527 patients with a diagnosis of schistosomiasis between 2013 and 2019, 21% had outpatient prescription drug claims reported for praziquantel. Patients with a diagnosis of schistosomiasis who were treated with praziquantel had different characteristics when compared with untreated patients (Table 1).

**Table 1:** Sample characteristics of patients with schistosomiasis diagnoses by praziquantel treatment status, 2013–19

Characteristic	Untreated (*n* = 414)	Treated (*n* = 113)	Total (*N* = 527)	*P* value
Gender, %				
Female	51.2 (2.5)	44.2 (4.7)	49.7 (2.2)	0.19
Male	48.8 (2.5)	55.8 (4.7)	50.3 (2.2)	
Age, y	40.7 (0.8)	38.4 (1.4)	40.2 (0.7)	0.15
≤ 40 years old, %	43.7 (2.4)	58.4 (4.7)	46.9 (2.2)	< 0.01
> 40 years old, %	56.3 (2.4)	41.6 (4.7)	53.1 (2.2)	
Living in rural area, %	11.1 (1.5)	1.8 (1.2)	9.1 (1.3)	< 0.01
No. of outpatient-visit days, d	1.3 (0.04)	2.0 (0.1)	1.4 (0.04)	< 0.01
Insurance type, %				
Capitated	14.0 (1.7)	8.8 (2.7)	12.9 (1.5)	0.15
Non-capitated	86.0 (1.7)	91.2 (2.7)	87.1 (1.5)	
Species of schistosome*, %				
*S. haematobium*	27.8 (2.2)	12.4 (3.1)	24.5 (1.9)	< 0.01
*S. mansoni*	16.4 (1.8)	13.3 (3.2)	15.7 (1.6)	
Others (specified and unspecified)	56.3 (2.4)	76.1 (4.0)	60.5 (2.1)	
Period, %				
2013–14	32.8 (2.3)	38.1 (4.6)	34.0 (2.1)	0.01
2015–16	37.2 (2.4)	22.1 (3.9)	34.0 (2.1)	
2017–19	30.0 (2.3)	39.8 (4.6)	32.1 (2.0)	

Standard errors are shown in parentheses. *P* values are from X^2^ test, and two-tailed Welch’s *t*-test with unequal variances.

* *Schistosoma haematobium* included *International Classification of Diseases, 9th Edition, Clinical Modification* (ICD-9-CM) code 120.0 and ICD-10-CM code B65.0. *Schistosoma mansoni* included ICD-9-CM code 120.1 and ICD-10-CM code B65.1. Others included ICD-9-CM codes 120.8 (other specified schistosomiasis) and 120.9 (schistosomiasis, unspecified), and ICD-10-CM codes B65.8 (other schistosomiasis) and B65.9 (schistosomiasis, unspecified).

Patients living in rural areas were less likely to be treated with praziquantel than patients living in urban areas (adjusted odds ratio [AOR] = 0.18; *P* = 0.02; Table 2). The odds of use of praziquantel among patients with ≥ 2 outpatient-visit days between 30 days before and 90 days after the first diagnoses of schistosomiasis was 4.1 times greater than those with only 1 outpatient-visit day during the same timeline (*P* < 0.01). Furthermore, patients with an *S. haematobium* infection diagnosis were less likely to receive praziquantel than those with “other specified or unspecified schistosomiasis” (AOR = 0.43; *P* = 0.01). Patients who were first diagnosed with schistosomiasis in 2015–16, after the dramatic price increase, were less likely to be treated with praziquantel than those who were first diagnosed in 2013–14 (AOR = 0.52; *P* = 0.03).

**Table 2:** Utilization of praziquantel among patients with schistosomiasis diagnoses, 2013–19

Characteristic	Adjusted odds ratio (95% CI)	*P* value
Gender		
Female	Reference	–
Male	1.31 (0.83–2.08)	0.24
Age		
≤ 40 years old	Reference	–
> 40 years old	0.69 (0.44–1.09)	0.12
Living area		
Urban	Reference	–
Rural	0.18 (0.04–0.80)	0.02
No. of outpatient-visit days		
1 day	Reference	–
≥ 2 days	4.10 (2.58–6.51)	< 0.01
Insurance type		
Non-capitated	Reference	–
Capitated	0.69 (0.33–1.48)	0.34
Species of schistosome*		
Others (specified and unspecified)	Reference	–
*S. haematobium*	0.43 (0.23–0.82)	0.01
*S. mansoni*	0.77 (0.40–1.51)	0.45
Period		
2013–14	Reference	–
2015–16	0.52 (0.29–0.93)	0.03
2017–19	1.03 (0.60–1.76)	0.92

* *Schistosoma haematobium* included *International Classification of Diseases, 9th Edition, Clinical Modification* (ICD-9-CM) code 120.0 and ICD-10-CM code B65.0. *Schistosoma mansoni* included ICD-9-CM code 120.1 and ICD-10-CM code B65.1. Others included ICD-9-CM codes 120.8 (other specified schistosomiasis) and 120.9 (schistosomiasis, unspecified), and ICD-10-CM codes B65.8 (other schistosomiasis) and B65.9 (schistosomiasis, unspecified).

Of 88 patients with a schistosomiasis diagnosis who were treated with praziquantel and with non-capitated insurance plans, mean total drug payments for praziquantel per patient increased from $110 (95% CI, $93–$126) in 2013–14 to $612 (95% CI, $510–$713) in 2015–18 (*P* < 0.01) after the dramatic increase in praziquantel AWP (Table 3). The increase in drug payments was statistically significant for insurance companies but not significant for patients’ out-of-pocket payments. In 2013–14, private insurance paid $50 per patient (95% CI, $33–$67) for praziquantel compared with $517 per patient (95% CI, $413–$621) in 2015–18 (*P* < 0.01). Changes in mean non-drug payments per patient were not statistically significant over the study periods. Total non-drug payments were $649 per patient (95% CI, $388–$910) in 2013–14 compared with $588 per patient (95% CI, $338–$839) in 2015–18 (*P* = 0.37).

**Table 3:** Mean outpatient drug and non-drug payments by payer over period among patients with schistosomiasis diagnoses who have praziquantel drug claims with non-capitated insurance plans, 2013–2018 (*N* = 88)

Payment by payer	2013–14 (n = 38)	2015–18 (n = 50)	*P* value*
Drug payment, US$, mean (95% CI)			
Insurance	50 (33–67)	517 (413–621)	< 0.01
Patient out of pocket	60 (46–73)	94 (50–139)	0.07
Total	110 (93–126)	612 (510–713)	< 0.01
Non-drug payment, US$, mean (95% CI)			
Insurance	534 (311–757)	475 (268–682)	0.35
Patient out of pocket	115 (47–184)	113 (57–170)	0.48
Total	649 (388–910)	588 (338–839)	0.37

* *P *values are from one-tailed Welch’s *t*-test with unequal variances.

The treatment costs of schistosomiasis infections and the use of praziquantel among patients with a diagnosis of schistosomiasis in non-endemic areas have not been well understood. We found that only one in five patients with a diagnosis of schistosomiasis in the United States received appropriate drug treatment. This was even less in rural areas, where many immigrants resettle because of job opportunities (e.g., meat-packing plants). Also, this rate is less than the utilization rates of standard-of-care drugs for soil-transmitted helminth infections in the United States reported previously.^7^

Reasons for the low use of praziquantel are unknown, but could be related to misdiagnosis, coding error, diagnosis of non-active infection (e.g., patients who were previously treated but continue to have persistent positive serology tests), failure to monitor a patient with a positive test, loss of follow-up, failure to fill the prescribed medication, or U.S. health providers’ unfamiliarity with diagnosis and management of schistosomiasis. The lower utilization rates of praziquantel during 2015–16, the period just after the dramatic increase in the drug price, compared with the utilization rates during 2013–14 suggest that the increase in drug price was associated with decreased use, even though patients did not experience significantly increased out-of-pocket payments. This finding is consistent with previous studies showing reduced use of antiparasitic drugs following large price increases.^7^^,^^8^ Also, the low use of praziquantel in rural areas emphasized the existence of rural health disparities and the need to increase utilization rates in rural areas.

There are limitations to this study. The population analyzed was limited to those with private insurance and, in particular, did not include uninsured individuals, who bear the full cost of treatment. A more than 350% drug price increase for uninsured individuals would be expected to decrease dramatically one’s ability to pay for standard-of-care drug treatment. It is very likely that these patients would have even less than 20% treatment rates. In addition, there are other limitations, such as missing data or coding errors inherent to the use of claims data, as well as the limited representativeness of the MarketScan^®^ database, as reported previously.^9^ Also, the MarketScan^®^ database does not include patients’ socioeconomic characteristics, such as income or education.

In summary, a large majority in the U.S. diagnosed with schistosomiasis did not have standard-of-care prescription drug claims. The dramatic price increase for praziquantel in 2014 further depressed the utilization rate even in this privately insured group of patients. Utilization rates would be expected to be even less for uninsured patients paying out of pocket, and, because of barriers, many more immigrants are uninsured than U.S. citizens.^10^ This disparity appears to be even greater in rural America, suggesting interventions and global health education to healthcare providers may benefit these communities.
